# Post-intensive Care Syndrome in a Heterogeneous Pediatric Population

**DOI:** 10.7759/cureus.32928

**Published:** 2022-12-25

**Authors:** Muthiah R Annamalai, Joshua T Kuehne, Kiran Mainali, Utpal Bhalala

**Affiliations:** 1 Pediatrics, University of Tennessee Health Science Center, Memphis, USA; 2 Medical School, University of the Incarnate Word School of Osteopathic Medicine, San Antonio, USA; 3 Epidemiology and Biostatistics, University of the Incarnate Word School of Osteopathic Medicine, San Antonio, USA; 4 Pediatrics, Texas A&M College of Medicine, College Station, USA; 5 Anesthesiology and Critical Care, University of Texas Medical Branch at Galveston, Galveston, USA; 6 Anesthesiology and Critical Care, Driscoll Children’s Hospital, Corpus Christi, USA

**Keywords:** functional status score (fss), pediatric intensive care unit (picu), outcomes, critical care, post-intensive care syndrome (pics), children

## Abstract

Background

In this study, we aimed to measure the incidence of post-intensive care syndrome among children (PICS-p) who received critical care treatment in our hospital and evaluate patient characteristics and critical care interventions associated with the development of PICS-p.

Methodology

We conducted a retrospective cohort review of all surviving pediatric patients admitted to an urban, academic, tertiary intensive care unit between July 2017 and June 2018. Based on the existing literature on PICS, we excluded children whose length of stay in the pediatric intensive care unit (PICU) was less than four days. We collected demographic data, clinical data, and data related to outcomes in our study cohort. We defined PICS-p a priori as a change in the Functional Status Scale (FSS) score of three or greater between pre-admission and discharge. Using Student’s t-tests and Wilcoxon rank-sum tests, we compared outcomes among those with PICS-p versus those without PICS-p.

Results

Of the 183 patients, 36 (19.6%) were diagnosed with PICS in our study. Aside from pre-admission FSS (7 vs. 8), analysis of the two groups revealed no statistically significant difference before or at the time of admission. Upon admission to the PICU, statistically significant differences between the PICS and no PICS groups were noted in the hospital length of stay (33.5 days vs. 14.7 days), ventilation-free days (8.3 days vs. 5.2 days), and the number of procedural interventions (2.6 vs. 1).

Conclusions

Utilizing the FSS to determine PICS is a viable method to standardize the measurement of functional outcomes for critically ill children. In our single-center, retrospective review, nearly one out of five pediatric patients developed PICS with associated factors that included a decreased pre-hospital FSS score, increased hospital length of stay, fewer ventilation-free days, and increased number of procedural interventions. Significant opportunities exist regarding the social and psychiatric domains of PICS-p.

## Introduction

The mortality rate has traditionally been the main measurement of outcomes in the intensive care unit. Significant strides made in the diagnoses and treatment of critically ill children in high-income countries have resulted in pediatric intensivists being far more likely to encounter functional impairments than mortality [1]. This increase in morbidity, combined with the shifting societal attitudes regarding the quality of life, has led to an increased emphasis being placed on functional outcomes in the pediatric intensive care unit (PICU) [2-4]. The creation of post-intensive care syndrome (PICS), defined by the 2010 Critical Care Stakeholder’s conference as a “new or worsening impairments in physical, cognitive, or mental health status arising after critical illness and persisting beyond acute care hospitalization,” is an attempt to better understand functional outcomes in a critically ill population and has since been modified for pediatrics [5,6].

Although many studies have been recently published addressing various aspects of functional impairments in survivors of intensive care, the existing framework of PICS in children (PICS pediatric, PICS-p) has not been utilized [7,8]. The lack of a standardized definition, along with the wide variety of measurement tools and the heterogenous nature of pediatric populations, have resulted in findings that are challenging to generalize or replicate [9]. One emerging tool to measure functional outcomes, the Functional Status Scale (FSS), is a validated and granular assessment developed by the National Institute of Child Health and Human Development (NICHD) Collaborative Pediatric Critical Care Research Network (CPCCRN) that can retrospectively measure functional outcomes of critically ill children of all ages [2]. Addressing two of the four PICS-p domains, physical and cognitive status, the FSS can investigate PICS in a more standardized fashion.

To our knowledge, no study has utilized FSS alone to measure the incidence of PICS-p. Using FSS, we aimed to define the incidence of PICS in all surviving pediatric patients who had received critical care treatment in our hospital. We also evaluated patient characteristics and critical care interventions associated with the development of PICS as measured by the FSS to compare these factors with associated factors and risk factors described in existing studies.

## Materials and methods

Patients

A retrospective chart review was conducted among all patients admitted to the PICU from July 2017 to June 2018 at The Children’s Hospital of San Antonio, Texas, USA, an urban, academic, tertiary center that admits both medical and surgical pediatric patients. Approval was obtained from the Institutional Review Board of Baylor College of Medicine. Patients were eligible for inclusion if they were under the age of 18, stayed four days or more in the PICU, and survived hospitalization. All patients who spent less than four days in the PICU were excluded as they had been shown to receive less critical care services proportionally, with patients admitted to the PICU for four days or greater constituting over 70% of total PICU days [2].

Data and outcomes

Electronic medical records of all eligible patients were reviewed for data on patient characteristics and hospital admission, including age, gender, primary organ dysfunction, underlying comorbidities, pre-admission FSS, PRISM, admission FSS, and surgery prior to admission. Primary organ dysfunction upon admission was categorized as neurologic, respiratory, cardiovascular, and other (ENT, gastrointestinal, genitourinary, musculoskeletal, hematology/oncology, and endocrinology) systems. The primary outcome was the difference between discharge FSS and pre-admission FSS. PICS was defined a priori as a change in the FSS score of three or greater from pre-admission to discharge. The definition was derived from the definition of significant functional impairment established by Heneghan et al. as a decline in FSS of 3 or more overall points [3]. Secondary outcomes included post-admission data, including PICU length of stay (days), hospital length of stay (days), admission FSS, ventilation-free days, number of procedural interventions, discharge FSS, and number of hospitalizations within a one-year period.

Statistical analysis

Multiple linear regression was used to analyze the relationship between change in FSS with all other independent factors in the overall cohort. One and two-tailed Student’s t-tests and Wilcoxon rank-sum tests were utilized to compare continuous data between patients with PICS and those without PICS. Pearson’s chi-square test, Fisher’s exact test, and odds ratios were used to compare categorical data between the two groups.

## Results

**Figure 1:**
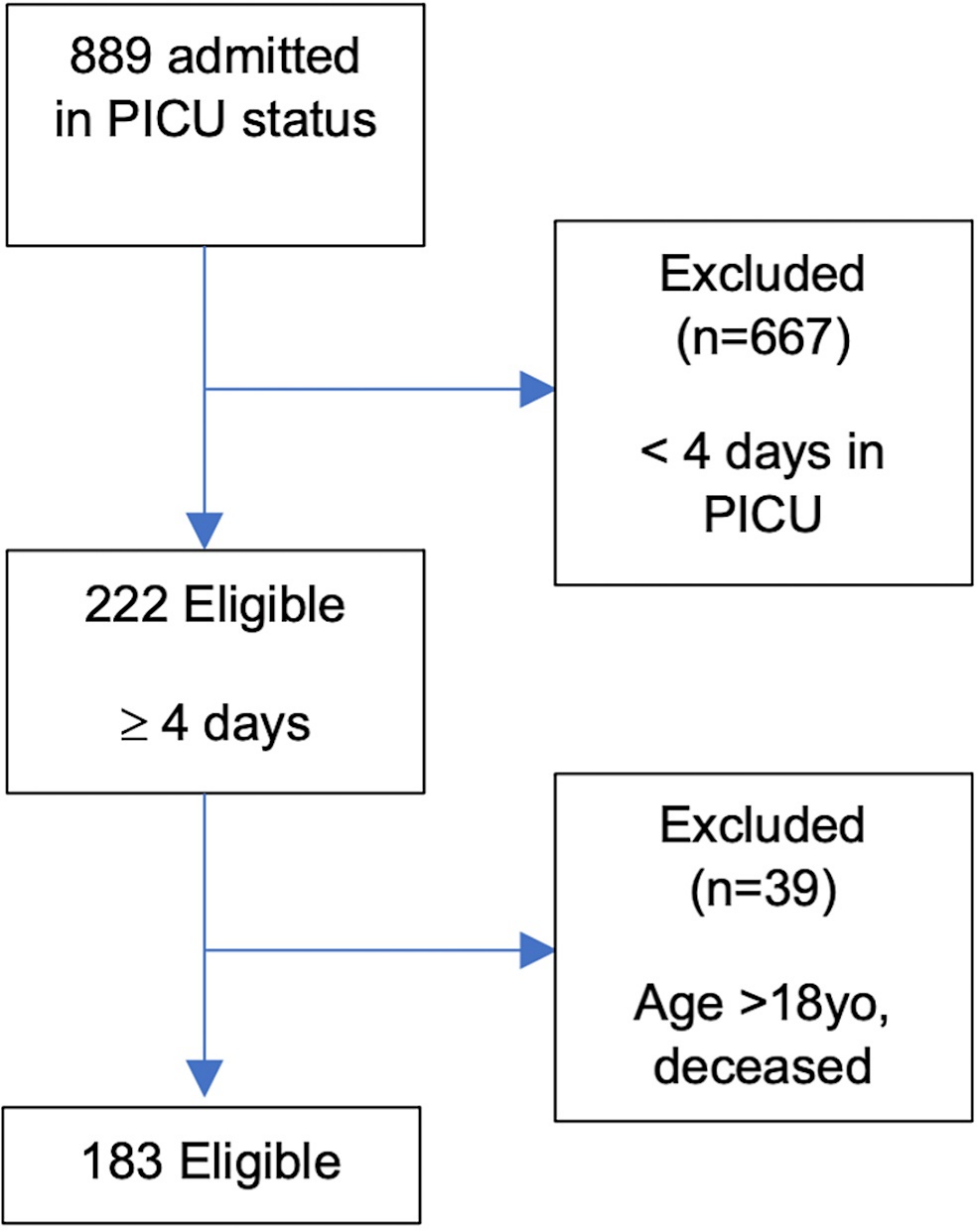
Consort diagram explaining the inclusion and exclusion of patients in our study and the final number of eligible patients in our study. PICU = pediatric intensive care unit; yo = year-old

During the study period, a total of 889 critically ill children were admitted to our PICU. Of these, 667 children were excluded due to their short (fewer than four days) length of stay in the PICU. Out of the remaining 222 children, 39 were excluded due to age >18 years and/or death during their PICU stay. Finally, 183 of 889 patients met the eligibility criteria (Figure 1).

The median (interquartile range, IQR) age of patients in our study was five (1-12) years. There was an equivalent representation of both genders in the cohort, with males and females constituting 51% and 49% of our cohort, respectively for a male-to-female ratio of 1.06:1. Overall, 48% of our cohort had two or more co-morbidities upon admission to the PICU. The most common primary organ dysfunctions in the overall cohort were the neurologic, respiratory, and cardiovascular systems. The pre-admission and discharge FSS (median (IQR)) were 6 (6-8) and 8 (6.5-10.5), respectively. Characteristics of the overall cohort are shown in Table 1.

**Table 1:** Demographic information and clinical characteristics of the overall cohort. IQR = interquartile range; FSS = Functional Status Scale

Demographic information and clinical characteristics	
Total patients	183
Age (years) (median (IQR))	5 (1-12)
Male/Female ratio	1.06:1
Primary organ dysfunction	
Neurologic (%)	27
Respiratory (%)	27
Cardiovascular (%)	24
Other (%)	22
Two or more comorbidities (%)	48
Pre-admission FSS (median (IQR))	6 (6-8)
Discharge FSS (median (IQR))	8 (6.5-10.5)

Outcomes

Using our definition of PICS a priori, 36 of 183 (19.6%) patients were diagnosed with PICS in our study and were subsequently compared to patients without the diagnosis (Table 2).

**Table 2:** Comparative analysis of patients with and without PICS based on FSS. **: statistically significant. PICS = post-intensive care syndrome; OR = odds ratio; IQR = interquartile range; FSS = Functional Status Scale; PICU = pediatric intensive care unit

	PICS (change in FSS ≥3)	No PICS (change in FSS <3)	P-value	OR
Total patients	36	147		
Age (years)			0.24	
Mean	5.3	7.3		
Median (IQR)	3 (0-10.5)	7 (1-12.5)		
Sex (female/male ratio)	1.12:1	0.9:1	0.58	1.23 (0.59-2.55)
Primary organ dysfunction			0.71	
Neurologic (%)	25	27		
Respiratory (%)	31	25		
Cardiovascular (%)	25	24		
Other (%)	19	24		
Two or more comorbidities (%)	39	49	0.28	1.15 (0.72-3.17)
Surgery prior to admission (%)	43	44	0.97	1.95 (0.88-4.34)
Pre-admission FSS			<0.001**	
Mean	7	8		
Median (IQR)	6 (6-6)	7 (6-9)		
Admission FSS			0.73	
Mean	20	19		
Median (IQR)	23.5 (11.75-27)	16 (12-27)		
PICU length of stay (days)			0.21	
Mean	20	19		
Median (IQR)	9 (7-14.5)	6 (5-10)		
Hospital length of stay (days)			<0.001**	
Mean	33.5	14.7		
Median (IQR)	35.5 (17.5-52.5)	8 (5-15)		
Ventilation-free days			<0.046	
Mean	8.3	5.2		
Median (IQR)	5 (2.75-9)	5 (3-6)		
Number of procedural interventions			<0.001**	
Mean	2.6	1		
Median (IQR)	2 (1-4)	1 (0-1)		

Aside from pre-admission FSS (7 vs. 8), analysis of the two groups revealed no statistically significant difference before or at the time of admission. Upon admission to the PICU, statistically significant differences between the PICS and no PICS groups were noted in hospital length of stay (33.5 days vs. 14.7 days), ventilation-free days (8.3 days vs. 5.2 days), and the number of procedural interventions (2.6 vs. 1).

## Discussion

This study utilized the FSS to investigate the incidence and associated factors of PICS in a heterogeneous group of patients undergoing prolonged PICU care. To our knowledge, this is the first study to use FSS alone to determine the incidence of PICS-p. Nearly one out of every five patients (19.6%) who stayed in our PICU for at least four days developed PICS, which is consistent with the incidence of PICS in adults and of functional impairments in children described in the literature [1,7,8,10-13]. The development of PICS in our population was associated with a lower pre-admission FSS score, a longer hospital length of stay, an increased number of ventilation-free days, and an increased number of procedural interventions. Of these associations, only the number of procedural interventions has been described as a risk factor in existing studies of functional impairments in critically ill children [8].

Unsurprisingly, children who developed PICS spent more time in the hospital, particularly in the setting of our institution’s established rehabilitation service. There was no significant difference in PICU length of stay between the two groups after analysis, which is consistent with existing literature and encourages a deeper investigation of the specific aspects during the PICU course that affect the development of PICS. While not significant, the longer length of stay in the PICU on average may explain why patients with PICS had increased ventilation-free days relative to patients without PICS. Without taking this into account, our data would contradict existing studies that describe mechanical ventilation as a risk factor for functional impairment [14]. The lower pre-admission FSS score of children who developed PICS may be a bias of using FSS, which only accounts for physical and cognitive impairments and not psychiatric or social impairments. Consequently, children who have a higher level of physical and cognitive impairments at baseline may not be properly evaluated for PICS. Surprisingly, our study diverged from current data by showing that younger age was not significantly associated with the rate of PICS, although the ages of patients who developed PICS were lower on average. Our study did not investigate the roles of socioeconomic status, education, or medications, all of which have been described as risk factors for functional impairment [10,14,15].

Significant limitations to our study arise by using FSS to measure PICS, as briefly eluded to above, as FSS only measures two of the four domains in the PICS-p paradigm. Consequently, the incidence of PICS in our study is likely underrepresented, particularly for children with pre-existing physical and cognitive deficits. Additionally, the level of nuance provided by FSS does not compare to the 25 validated tools that currently exist to quantify the various aspects of PICS [9]. The benefit of FSS, however, is a standardized and validated method to measure the incidence of PICS; currently, the incidence of impairments in physical function (4-81%), cognitive function (3-42%), mental health (13-83%), and social health (9-21%) vary tremendously based on the utilized tool. Additional limitations include the retrospective nature of our study, the lack of follow-up, the sample size, and the measurement of illness severity. The retrospective framework used to investigate PICS predisposes our results to documentation biases and limits the possible conclusions regarding associated factors. The retrospective review also results in a lack of standardized follow-up needed to measure the persistence of PICS with time. The single-center sample size of the study is a limiting factor, particularly as the incidence of PICS ranges widely in existing studies. Moreover, we did not validate the diagnostic criteria of PICS-p, and, therefore, it is possible that the change in FSS of 3 points may be too sensitive to diagnose PICS-p in our study. Though we used a prior study by Heneghan et al. and defined PICS-p a priori based on the change in FSS by 3 or more from baseline, future studies are needed to validate the diagnostic criteria of PICS-p using FSS. It is possible that our inclusion criterion of a four-day or longer PICU stay was associated with selection bias. Finally, the lack of our electronic medical record to calculate PRISM III scores poses a significant confounding factor as illness severity has been described as a risk factor for the incidence of PICS in existing literature [8].

Future directions of research include determining the persistence of PICS after discharge using FSS. Additionally, creating validated tools to retrospectively review social and psychiatric domains for critically ill children will help describe the incidence of PICS in a standardized fashion. FSS can also be utilized to measure the impact of medications, nutrition, therapy services, and other aspects of the PICU on the incidence of PICS. Finally, more multicenter, prospective studies that utilize FSS to measure PICS will help identify generalizable factors that impact its incidence.

## Conclusions

Based on FSS, our single-center, retrospective review found that nearly one out of five pediatric patients developed PICS. In our cohort, decreased pre-hospital FSS score, increased hospital length of stay, fewer ventilation-free days, and increased number of procedural interventions were associated with PICS. Significant opportunities exist regarding the social and psychiatric domains of PICS-p.
